# Cognition in Patients With Multiple System Atrophy (MSA) and Its Neuroimaging Correlation: A Prospective Case-Control Study

**DOI:** 10.7759/cureus.21717

**Published:** 2022-01-29

**Authors:** Santosh Dash, Rohan Mahale, M. Netravathi, Nitish L Kamble, Vikram Holla, Ravi Yadav, Pramod K Pal

**Affiliations:** 1 Neurology, Kalinga Institute of Medical Sciences, Bhubaneswar, IND; 2 Neurology, National Institute of Mental Health and Neurosciences, Bangalore, IND; 3 Neurology, National Institute of Mental Health and Neuro Sciences, Bengaluru, IND

**Keywords:** msa-c, msa-p, voxel-based morphometry, cognition, multiple system atrophy

## Abstract

Objective

Cognition has been reported to be involved in patients with multiple system atrophy (MSA), although initially it was considered an exclusion in the diagnosis of MSA. We assessed cognition in these patients and compared it with age and education matched healthy controls and correlated with the gray matter volume using voxel-based morphometry (VBM).

Materials and methods

This was a prospective, case-control, single-center study. Thirty patients with MSA (20 MSA-C (cerebellar variant) and 10 MSA-P (Parkinsonian variant)) and 25 age- and educational level-matched healthy controls were included. All the patients and controls underwent detailed neuropsychological tests and MRI brain. A battery of neuropsychological tests like Stroop test, digit span forward and backward, digit symbol substitution time test, animal naming test, color trail test and auditory verbal learning test were used to assess the various domain of cognition, which include mainly attention, executive function, memory, new learning, mental and motor speed. The gray matter volume was determined using VBM and correlated with neuropsychological scores.

Results

Attention, execution, verbal and visual memory, verbal fluency, and new learning were impaired in patients with MSA. MSA-P had more impairment in motor and mental speed, working memory, executive functions, and focused attention compared to MSA-C. Patients with MSA-C had more impairment in new learning, immediate recall, verbal fluency, and sustained attention compared to MSA-P. However, it was not statistically significant. There was a significant correlation between the various cognitive domains and atrophy of frontotemporal cortical areas, insula, caudate, thalamus, and cerebellum.

Conclusion

Cognition is impaired in patients with MSA-C and MSA-P and is likely due to the neurodegenerative process involving the cortical and subcortical structures. Long-term follow-up studies are required to find out the progression of these cognitive changes.

## Introduction

Multiple system atrophy (MSA) is an adult-onset sporadic neurodegenerative disease clinically defined by severe autonomic failure, parkinsonism, and/or cerebellar ataxia [1]. It is the second most common neurodegenerative movement disorder with an average annual incidence rate of 3 per 100000 person-years after Parkinson’s disease (PD) [2]. It is sub-classified into a parkinsonian (MSA-P) and a cerebellar variant (MSA-C) based on the predominant motor phenotype. Similar to the non-motor symptoms (NMS) in patients with PD, patients with MSA have been recognized to harbor these symptoms. Cognition is also involved in patients with MSA [3]. However, dementia was considered a non-supporting feature in the second consensus criteria for diagnosing MSA [4]. The severity of cognitive impairment in MSA reported so far ranges from mild to moderate [5]. The prevalence rates of mild, moderate, and severe cognitive impairment in autopsy-confirmed MSA are 22%, 2%, and 0.5%, respectively [3]. The degree of motor impairment was established as a predictor of cognitive impairment in MSA [6]. Cognitive impairment in MSA is due to the accumulation of glial cytoplasmic inclusion (GCI) in cortical layers of the frontal, parietal, temporal, and cingulate cortex [7, 8]. It has been postulated to be due to the α-synuclein deposition in the striatonigral system disrupting the frontal subcortical circuits along with hypoperfusion in the dorsolateral prefrontal cortex [9]. There is a paucity of literature about cognition in MSA from the Indian subcontinent. The study aimed to assess cognition in patients with MSA and its structural correlation using voxel-based morphometry (VBM).

## Materials and methods

Study design

This was a prospective, case-control, single-center study conducted in the departments of Neurology, Clinical Psychology, and Neuroimaging and Interventional Radiology at the National Institute of Mental Health and Neuro Sciences (NIMHANS), Bangalore, India. The study was approved by the Institute Ethics Committee (IEC/No.3.02/3-11-2015) and written informed consent was obtained from all the study participants. 

Study subjects

The study included 30 patients with MSA (20 MSA-C and 10 MSA-P) diagnosed according to the second consensus diagnostic criteria for MSA [4]. Patients whose mini-mental state examination (MMSE) score was less than 24 and those with major systemic illness were excluded. Twenty-five age- and education-matched healthy controls were included. All the subjects underwent detailed neuropsychological evaluation using the NIMHANS Neuropsychology battery and MRI brain. 

Data collection

Neurological Assessment

The following data were collected from the study participants: sociodemographic details - age at presentation, age of onset, gender, duration of illness, educational qualification, socioeconomic status, marital status, and comorbid illness; clinical data - details about parkinsonian symptoms such as tremor, bradykinesia, rigidity, postural instability, and levodopa responsiveness, cerebellar symptoms like gait or limb ataxia, speech disturbances, etc. The severity of MSA was assessed by the Unified Multiple System Atrophy Rating Scale (UMSARS) (permission obtained from the Movement Disorders Society) [10]. The following NMSs were assessed: sleep quality using Pittsburgh sleep quality index scale (PSQI), which consists of seven components, i.e., subjective sleep quality, sleep latency, sleep duration, habitual sleep efficiency, sleep disturbances, drugs intake for sleep, and daytime dysfunction over the last month, with a score of 5 or above considered as poor sleepers [11], rapid eye movement (REM) sleep behavior disorder (RBD) using REM Sleep Behavior Disorder Screening Questionnaire (RBDSQ), with a score of 5 or above suggestive of RBD [12], anxiety and depression using Hospital Anxiety and Depression Scale (HADS) [13].

Cognitive Assessment

MMSE was used as the screening tool for inclusion in the study [14]. Those patients having an MMSE score of more than 24 underwent detailed neuropsychological tests. Detailed cognitive functions were assessed using tests from NIMHANS adult neuropsychological battery. The tests were used to assess motor and mental speed, attention, fluency, working memory, learning, delayed memory, and executive functions. The motor speed was assessed using finger tapping, attention and visuoperceptual functions using digit symbol substitution test, attention using color trails 1 (CT-1), digit span forward and spatial span forward, sustained attention and psychomotor speed using digit vigilance test and learning and memory by Rey auditory verbal learning test (RAVLT). The core executive function assessed were response inhibition, cognitive or mental flexibility, and working memory. The Stroop test measures the response inhibition of subjects and consists of three parts Stroop words, Stroop color, and Stroop effect. The Stroop effect is the difference in the time taken for completing the Stroop color and Stroop word tests. Verbal working memory was assessed by digit span backwards and visual-spatial working memory by spatial span backwards. Mental flexibility was assessed with CT 2 test and animal naming test for verbal fluency.

MRI Data (Voxel-Based Morphometry)

The MRI data were acquired in Philips Achieva 3-T MRI system (Philips Healthcare, Best, Netherlands). MRI data analysis was done on voxel-based morphometry (VBM8) toolbox in SPM8 software (The Wellcome Centre for Human Neuroimaging, London, UK) using MATLAB R2013a (MathWorks, Natick, USA). The raw T1-weighted anatomical data of all the subjects in Digital Imaging and Communications in Medicine (DICOM) format were imported to SPM8 and saved as Statistical Parametric Mapping (SPM)-compatible NiFTI file format. Before preprocessing, all the subject’s data were manually reoriented to their respective anterior commissure-posterior commissure (AC-PC) plane. VBM involves a voxel-wise comparison of the local concentration of gray matter between two groups of subjects. The procedure involves pre-processing, smoothing, and statistical analysis. Pre-processing involved spatially normalizing the reoriented T1-weighted anatomical images from all the subjects in both the groups in the study into the same stereotactic space. The normalized images were segmented into gray matter (GM), white matter, cerebrospinal fluid (CSF), and non-CSF components. Segmented images were then spatially normalized to Montreal Neurological Institute (MNI) space and were modulated. Smoothing was applied on modulated images using an 8-mm full width half maximum (FWHM) Gaussian kernel for subsequent statistical analysis. 

Statistical analysis

All the clinical, neuropsychological data were analyzed using SPSS Statistics software version 22 (IBM Corp, Armonk, USA). The data were expressed using mean, standard deviation (SD) for continuous variables, and frequency and percentage for categorical variables. The comparison between MSA and control groups was done by independent sample t-test or Wilcoxon rank-sum test depending upon the normality of data. Comparison between MSA-C, MSA-P, and healthy controls was done by ANOVA/Kruskal-Wallis test. Correlation between clinical variables, and neuropsychology parameters was done by using Pearson/Spearman correlation coefficient. Categorical variables were analyzed by Chi-square test. P<0.05 was considered statistically significant. Multiple regression analysis was used for the correlation of clinical and neuropsychology scores with gray matter volume in patients. FWE correlation was applied with p<0.05. However, to convey a more complete picture of data, gray matter areas that may have a meaningful correlation with the various neuropsychological and clinical scores but not surpassing our statistical threshold we consider p<0.001 (uncorrected for multiple comparisons) as statistically significant

## Results

Thirty patients of MSA and 25 age-, gender-, and education-matched healthy controls were included. There was no difference between the MSA subtypes with respect to age at presentation, age at onset, gender, and duration of the disease. The sociodemographic profile and clinical characteristics of the patients and controls are summarized in Table 1.

**Table 1:** Comparison of demographic profile and clinical scales between MSA patients and controls p<0.05; ^a^mean & standard deviation; RBD-REM sleep behavior disorder; PSQI-Pittsburgh sleep quality index scale; MMSE-mini-mental state examination; UMSARS-Unified Multiple System Atrophy Rating Scale; SBP-systolic blood pressure; DBP-diastolic blood pressure; MSA-p-multiple system atrophy parkinsonian variant; MSA-C-multiple system atrophy cerebellar variant *p<0.001 statistically significant

	Patients (n=30)	MSA-P (n=10)	MSA-C (n=20)	Controls (n=25)	p-value
Age (years)^a^	54.4 ± 5.8	55.7 ± 5.4	53.8 ± 6.0	55.0 ± 6.8	0.74
Age at onset (years)^a^	51.8 ± 5.9	53.3 ± 4.7	51.0 ± 6.4	-	0.33
Gender (M/F)	17/13	5/5	12/8	18/7	0.23
Education (years) (median and range)	12 (8-18)	11 (9-18)	13 (7-18)	12 (7-18)	0.57
Duration of illness (years)^a^	2.6 ± 1.2	2.4 ± 1.3	2.7 ± 1.2	-	0.49
SBP drop ^a^	17.1± 11.8	16.4 ± 12.2	17.5 ± 11.9	-	0.38
DBP drop ^a^	7.9 ± 4.5	7.5 ± 2.3	8.2 ± 5.3	-	0.45
UMSARS part 1 ^a^	15.9 ± 8.4	15.4 ± 5.0	16.2 ± 9.8	-	0.84
UMSARS part 2 ^a^	16.0 ± 8.2	16.8 ± 7.7	16.9 ± 9.6	-	0.92
Anxiety score ^a^	7.2 ± 3.4	6.5 ±3.1	7.6 ± 3.6		0.08
Depression score ^a^	9.5 ± 3.6	9.6 ± 3.8	9.4 ± 3.5	-	0.44
Global PSQI score ^a^	9.1 ± 4.0	7.8 ± 4.1	9.7 ± 3.8	-	0.21
RBD (n/%)	19 (63)	4 (40)	15 (75)	-	0.06
RBD score (median and range)	5 (1-10)	3.5 (2-7)	7 (1-10)	-	0.09
MMSE score	27.8 ± 3.2	-	-	29.6 ± 0.6	< 0.001*
MMSE score	-	27.7 ± 1.8	27.8 ± 1.8	-	1.0

Clinical data

Gait ataxia was the most common initial symptom in 43.3% of patients. Other initial symptoms were slowness of activities in 16.7%, asymmetric tremors of hands in 16.7%, urinary disturbances in 6.7%, speech disturbances in 6.7%, and incoordination of hands in 3.3% of cases. RBD as an initial symptom was present in 6.7% of cases. In patients with MSA-C, gait imbalance was the most common initial symptom in 65 % of cases. Other initial symptoms were bladder dysfunction in 10 %, sleep disturbances in 10%, hand incoordination in 5%, speech disturbances in 5%, and slowness in 5%. In patients with MSA-P, asymmetric onset tremors of hands were the most common initial symptoms in 50% of cases, followed by the slowness of activities in 40% and speech disturbances in 10%. Urinary disturbances in the form of frequency and urgency were the most common autonomic dysfunction seen in 29 (96.7 %) patients. Orthostatic symptoms were seen in 10 (33.3%) patients (in 40% of MSA-C and 20% of MSA-P patients). Orthostatic hypotension occurred in 9 (30%), erectile dysfunction in 9 (53%) out of 17 male patients, constipation in 18 (60%) and hypohydrosis in 2 (7%) patients. Spasticity, brisk tendon jerks noted in 90% of cases with equal frequency in MSA-P and MSA-C and falls in 13.3 % of cases, predominantly in MSA-C.

Clinical scales

 UMSARS

There was no significant difference between MSA subtypes with respect to the mean UMSARS score part 1 and part 2. The median global disability score of MSA patients was 2 (1-5) with an equal score of 2 (1-5) in both MSA subtypes. 

HADS, RBDSQ, PSQI, MMSE

Anxiety symptoms were noted in 13 (43.3%) patients and depression in 24 (80%) patients. Sleep disturbances were present in 24 (80%) patients. Prolonged sleep latency was the most common sleep abnormality found in 60% of patients. Sleep disturbances were seen in 18 (90%) MSA-C patients and in 6 (60%) MSA-P patients. In the patient group, the mean global PSQI score was 9.1 ± 4.0. RBD was present in 19 (63.3%) patients. Fifteen (75%) patients with MSA- C and 4 (40%) patients with MSA-P had RBD. The mean MMSE score was significantly lower in patients (Table 1). 

Neuropsychological Assessment

There was a significant difference in the mean finger taps of both hands between patients and controls with no difference between the subtypes. The digit symbol substitution time, CT 1 and CT 2-time, digit vigilance time was significantly longer in patients with no difference between the subtypes. The animal naming score, digit span forward and backward score, spatial span forward and backward score was significantly low in patients with no difference between the subtypes. The total correct response of auditory verbal learning test (AVLT) was lower, the immediate and delayed recall was slower in patients with no difference between the subtypes. The Stroop word, Stroop color, and Stroop effect were significantly higher in patients with no difference between the subtypes. The clinical scales and neuropsychological scores of the patients and controls are summarized in Table 2.

**Table 2:** Comparison of neuropsychological parameters between MSA patients and controls p<0.05; ^a^mean and standard deviation; ^b^median; DST-digit symbol substitution time; DVT-digit vigilance test; AVLT-auditory verbal learning test; MSA-p-multiple system atrophy parkinsonian variant; MSA-C-multiple system atrophy cerebellar variant *p<0.001 statistically significant

Tests	MSA (n=30)	MSA-P (n=10)	MSA-C (n=20)	Controls (n=25)	P-value (patients vs controls)	P-value (MSA-P vs MSA-C)
Right finger taps ^a^	41.9± 13.0	41.6± 15.3	42.1±12.1	56.4 ± 4.8	<0.001 *	0.41
Left finger taps ^a^	36.3± 11.7	36.3± 10.4	36.4± 12.5	51.8± 4.13	<0.001 *	0.61
DST seconds ^b^(range)	202.0 (166-285)	389(245 -720)	364.5 (168 -960)	378 (168-960)	<0.001 *	0.75
Color trail-1 ^a^	114.0± 33.3	93.6 ± 27.2	124.2±31.8	54.5 ± 9.6	<0.001*	0.42
Color trail-2 ^a^	240.1± 72.5	248.0±73.4	236.2±73.7	106.2±12.6	<0.001*	0.81
DVT seconds ^b^(range)	643 (396-1440)	744 (490-1127)	623 (396-1440)	412 (340-490)	<0.001*	0.45
Animal naming ^a^	9.9 ± 2.1	10.7 ± 2.6	9.5 ± 1.8	16.6 ± 2.3	<0.001*	0.50
Digit span Forward ^b^(range)	4.0 (4-5)	4 (4-5)	4.5(4-5)	5 (4-7)	<0.001*	1.0
Digit span backward^ b^(range)	3 (2-4)	2.5 (2-3)	3(2-4)	4 (2-5)	<0.001*	0.77
Spatial span forward (range) ^b^	4(4-5)	4 (4-5)	4(4-5)	6 (4-7)	<0.001*	1.0
Spatial span backward (range)^ b^	3.5(2-4)	3 (3-4)	4(2-4)	5 (3-6)	<0.001*	1.0
Immediate recall (range)^ b^	8.5(4-11)	9(7-11)	8(4-11)	(10-14)	<0.001*	1.0
Delayed recall (range)	6(2-10)	6(5-10)	6(2-9)	(7-14)	<0.001*	1.0
Total correct AVLT ^a^	36.7± 6.5	38.2 ± 7.1	36 ± 6.1	50(43-59)	<0.001*	0.92
Stroop word (range) ^b^	135 (68-360)	137 (112-351)	130(68-360)	82 (58-126)	<0.001*	1.0
Stroop color (range) ^b^	342.5 (135-720)	358 (266-575)	330 (135-720)	186 (137-356)	<0.001*	0.85
Stroop effect (range) ^b^	197 (67-613)	206.5 (146-270)	189.5(67-613)	108 (78-140)	<0.001*	0.32

Correlation Between Clinical Parameters and Disease Severity

The duration of the illness and disease severity as per UMSARS-2 and global disability score [(r=0.14, p<0.01); (r=0.26, p<0.02)] had a significant positive correlation. The MMSE score had negative correlation with disease severity [(r=-0.35, p<0.05); (r=-0.53, p<0.002)]. Anxiety (HADS), depression (HADS), RBDSQ score had positive correlation with UMSARS-2 score (r=0.5, p<0.004; r=0.44, p<0.002; r=0.21, p<0.04). 

Correlation Between Neuropsychology and Clinical Parameters

There was a significant negative correlation between disease severity (UMSARS 1 and 2) and global disability with motor speed of right hand [(r=-0.55, p<0.001) (r=-0.63, p<0.001) (r=-0.69, p<0.001)] and left hand (r=-0.51, p<0.003) suggesting the reduction of motor speed occurred with an increase in the disease severity. The global disability and verbal fluency score had a negative correlation (r=-0.1, p<0.001), implying higher the disability poorer was the verbal fluency. A positive correlation was observed between UMSARS-2 and CT-2 score, Stroop effect (r= 0.28, p<0.001; r= 0.26, p<0.001) which suggested that worsening motor disability was associated with poor mental flexibility, response inhibition, and conflict resolution. A significant negative correlation was seen between UMSARS-2 and digit span forward score (r=-0.15, p<0.001), suggesting higher the motor disability, the lower the attentional abilities. There was a negative correlation between duration of illness and spatial span forward scores (r=-0.53, p=0.002) suggesting reduced attention with the progression of the disease. The delayed recall had a negative correlation with UMSARS 2 score (r=-0.06, p=0.001) which suggested that a higher motor disability was associated with a poor delayed recall. This is summarized in Table 3.

**Table 3:** Correlation of neuropsychological scores with clinical severity. p<0.005 is significant; FW-forward; BW-backward; AVLT-auditory verbal learning test; UMSARS-Unified Multiple System Atrophy Rating Scale

Neuropsychology	Age		Duration		UMSARS-1		UMSARS-II		Global disability		
	r	P	r	p	r	P	R	p	r	p	
Right Finger tap	-0.14	0.44	-0.05	0.65	-0.55	0.001	-0.63	0.001	-0.69		0.001
Left finger tap	-0.10	0.59	-0.16	0.94	-0.32	0.07	-0.37	0.04	-0.51		0.003
Color Trail -1	0.18	0.32	0.007	0.44	0.03	0.84	0.03	0.87	0.17		0.36
Color trail-2	0.07	0.69	-0.08	0.80	0.26	0.16	0.28	0.001	0.28		0.12
Animal naming	-0.06	0.74	-0.24	0.93	0.03	0.86	0.007	0.97	-0.10		0.001
Dig span FW	0.008	0.96	0.26	0.16	-0.06	0.73	-0.15	0.001	-0.11		0.54
Digit span BW	-0.29	0.11	0.03	0.85	-0.23	0.20	-0.27	0.14	-0.27		0.14
Spatial span FW	-0.29	0.26	-0.53	0.002	0.14	0.45	0.05	0.77	0.009		0.96
Spatial span BW	-0.17	0.35	-0.1	0.57	-0.23	0.21	0.01	0.92	-0.05		0.76
AVLT total correct	-0.16	0.37	-0.03	0.85	-0.07	0.70	0.02	0.89	0.04		0.79
Immediate recall	-0.32	0.07	-0.03	0.84	0.08	0.64	0.06	0.74	-0.04		0.81
Delayed recall	-0.35	0.05	-0.02	0.89	0.01	0.92	-0.06	0.001	-0.16		0.38
Stroop word	0.23	0.21	0.39	0.03	0.07	0.67	0.13	0.49	0.24		0.19
Stroop color	0.42	0.01	0.17	0.35	-0.01	0.93	0.11	0.55	0.13		0.49
Stroop effect	0.31	0.09	0.05	0.78	0.15	0.41	0.26	0.001	0.17		0.34

Correlation of Neuropsychology Parameters With Gray Matter Volume

**Figure 1:**
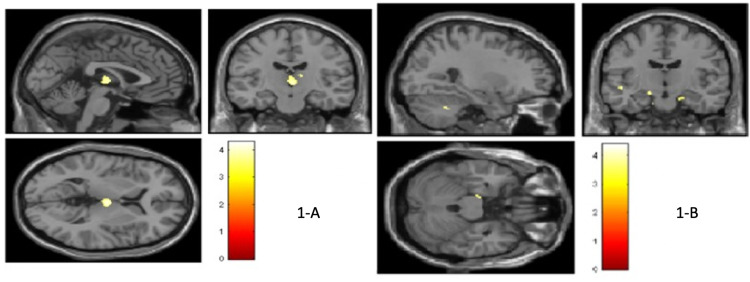
Voxel-based morphometry analysis (A) showing areas of gray matter having negative correlation with Stroop effect in MSA patients; (B) showing areas of gray matter having positive correlation with immediate recall score in MSA patients. MSA-multiple system atrophy

The Stroop effect and GM volume in the right precentral gyrus, medial dorsal nucleus of the thalamus, and insula (p<0.001 FWE uncorrected) had a significant negative correlation in the patient group (Figure 1A). The animal naming scores and GM volume in left medial frontal gyrus, right side precentral gyrus and precuneus (p<0.001 FWE uncorrected), digit span forward score with GM volume in left middle frontal gyrus, i.e., dorsolateral prefrontal cortex (DLPFC) (p<0.001 FWE uncorrected), the total correct response from RAVLT score and GM volume in right medial frontal gyrus, bilateral superior frontal gyrus and left side paracentral lobule (p<0.001 FWE uncorrected), delayed recall score with GM volume in the bilateral caudate head, left side cingulate and parahippocampal gyrus (p<0.001 uncorrected) had a significant positive correlation in the patients. Immediate recall score from RAVLT had a positive correlation with GM volume in right cerebellar tonsil, bilateral uncus, parahippocampal gyrus (p<0.001 FWE uncorrected) in patients (Figure 1B). This is summarized in Tables 5-10 (Appendices).

## Discussion

Several studies have shown a broad spectrum of deficits in cognition in MSA [15, 16]. Frontal-executive dysfunction is the commonest cognitive dysfunction affecting approximately up to 49% of MSA patients. These include problems with semantic and phonemic word list generation, perseverative behaviour, and impairments in problem-solving ability. Other cognitive domains like memory, visuospatial and constructional functions are also involved [17]. The frequency of cognitive impairment (CI) ranges from 22% to 37% in autopsy-confirmed MSA [18]. A more pronounced CI with MMSE ≤24 points indicating dementia was present in 7.4% of cases of MSA. This study was aimed at assessing the presence, pattern, and structural correlates of CI in MSA. We assessed the cognitive functions in 30 MSA patients and compared them with healthy controls. 

The cognitive profiles vary between the MSA subtypes. Executive dysfunction was reported in 40% of MSA-P patients which includes impairment in the speed of thinking and problem-solving, attentional set shifting, mental flexibility, abstract reasoning, and perseverative tendencies [19]. The commonest CI seen in MSA-C is executive dysfunction in up to 50 % of cases followed by moderately reduced verbal fluency [20,21]. However, there is a difference in the involvement of cognitive domains in the MSA subtypes. Visuospatial and constructional function, new learning has been reported to be impaired in both subtypes. The various studies on the cognitive profiles in MSA have been summarized in Table 4.

**Table 4:** Summary of studies on the cognitive deficits in MSA subtypes. MSA-multiple system atrophy

Studies	Group	Conclusion
Robbins et al [21]	MSA (n=16) vs controls (n=16)	MSA patients performed worse on spatial working memory task and conditional visuospatial associative learning tests
Bürk et al [20]	MSA-C (n=20) vs controls (n=20)	Impaired verbal memory and execution in MSA-C. Executive dysfunction due to the involvement of cerebrocerebellar circuits.
Kawai et al [22]	MSA-P (n=14), MSA-C (n=21) vs controls (n=21)	MSA-P patients had severe involvement of visuospatial and constructional function, verbal fluency, and executive functions. MSA-C patients had involvement only of visuospatial and constructional functions
Chang et al [16]	MSA-P (n=13) vs MSA-C (n=10)	MSA-C had more impairment of executive functions than MSA-P
Balas et al [23]	MSA-P (n=15) vs MSA-C (n=10)	MSA-P had reduced immediate and long-term verbal retrieval and MSA-C had difficulties in learning new verbal information and in attention
Hong et al [18]	MSA-C (n=26) vs controls (n=26)	Visuospatial function, 3 words recall, verbal immediate, delayed and recognition memory and visual delayed memory impaired in MSA-C
Kim et al [19]	MSA-P (n=15) vs controls (n=32)	Attention, memory recall, verbal fluency and frontal executive domains impaired in MSA-P
Barcelos et al [24]	MSA-P (n=10) vs MSA-C (n=4)	MSA-P and MSA-C had impaired executive and visuospatial functions; attention deficit was predominant only in MSA-C
Eschlbock et al [25]	MSA-P (n=39) vs MSA-C (n=15)	Executive function and verbal memory impaired in MSA. MSA-C patients performed significantly worse than MSA-P in the executive functions and in phonemic verbal fluency

In our study, patients with MSA-P had more impairment in motor and mental speed, working memory, executive functions, and focused attention than MSA-C but did not meet statistical significance. New learning, immediate recall, verbal fluency, and sustained attention were more impaired in MSA-C than MSA-P but did not meet statistical significance. Kawamura et al [15] and Hong et al [18] showed significant impairment of MMSE score in MSA subtypes. We found significantly lower MMSE scores in patients and had a negative correlation with disease severity suggesting progressive cortical and subcortical atrophy as the disease progressed leading to CI. The utility of the MMSE scale for assessment of cognition in MSA needs confirmation in the larger cohort of patients. 

Bürk et al [20] and Kawai et al [22] did not find any significant difference in attention scores between MSA patients and controls. We found impaired attention in both the subtypes of MSA. Verbal fluency and visual memory impairment have been reported in MSA [16, 23]. We found impaired verbal fluency, verbal and visual memory in our patients with MSA. Balas et al [23] reported worse performance in AVLT test by MSA patients. However, Siri et al [17] did not find any significant difference in learning between MSA subtypes. We found impaired learning, immediate and delayed recall abnormalities in MSA patients with no difference between the subtypes. Pillon et al [26] showed a normal Stroop test in MSA patients. We found impaired Stroop test in MSA patients with no difference between the subtypes. 

The effect of disease severity and disease duration on cognition was assessed by correlating the neuropsychiatric tests scores with the disease severity. The disease severity had a negative correlation with motor speed, working memory, verbal fluency, and a positive correlation with attention and executive function suggesting progressive cognitive impairment with the disease progression. The disease duration negatively correlated with visual memory scores suggesting reduced visual memory with increasing disease duration. Krishnan S et al (2006) reported a significant negative correlation between the motor scores of parkinsonism and cognitive function suggesting worsening cognitive functions with an increase in the disease severity in MSA [27].

The correlation of cognitive domains with gray matter volume has been studied in MSA. Significant loss of gray matter volume in the frontal lobe has been reported in MSA patients with attentional impairment [28]. Lee et al [29] showed that the attention deficit in MSA-C was related to the atrophy in the left calcarine gyrus and cerebellum, executive and visuospatial dysfunction were related to the atrophy in the thalamus. Chang et al [16] reported correlation atrophy of left superior and inferior frontal region with verbal and non-verbal episodic memory impairment. We found a positive correlation of attention and verbal memory with reduced GM volume in the left dorsolateral prefrontal cortex (DLPFC), a significant negative correlation of Stroop effect with GM volume in the right precentral gyrus in the frontal lobe, right insula, and thalamus indicating impaired attention, verbal memory and executive function is associated with reduced GM volume in the frontal lobe, insula, and thalamus. The role of the insula in executive dysfunction may be due to the loss of efferent pathways from the insula to the prefrontal cortex. Caso F et al [30] showed a correlation between the Addenbrooke’s Cognitive Examination-Revised (ACE-R) memory subscale scores and cortical thinning in bilateral superior temporal gyrus (STG), right temporal pole and fusiform gyrus, between ACE-R global scale scores and cortical thinning in right STG and parahippocampal gyrus; and between MMSE scores and cortical thinning of the cingulate cortex. Our study showed that the new learning had positive correlation with GM volume of bilateral superior frontal gyrus (Area 6), right side middle frontal gyrus, and left side paracentral lobule. Immediate recall score had a positive correlation with GM volume in left-side uncus, bilateral parahippocampal gyrus, and right-side cerebellum. Delayed recall score had a positive correlation with GM volume in the bilateral caudate body, left-side cingulate gyrus (Area 24), and left parahippocampal gyrus (Area 28). The temporal lobe has a significant role in cognition. Visuospatial processing and episodic memory are associated with the parahippocampal cortex, phonological processing, and audiovisual/ audiomotor speech integration by STG and executive functioning, memory, emotion processing, and social cognition by cingulum. 

The neuropathological underpinnings of cognitive deficits in MSA have not been completely understood. Degeneration of subcortical structures causes disruption of circuits from the frontal cortex to basal ganglia and thalamus leading to cognitive deficits. Intrinsic cortical pathology in the form of neuronal cytoplasmatic inclusions in the neocortex or limbic regions, thinning of neocortices on imaging have been associated with cognitive impairment in MSA [7]. We found that executive dysfunction, attention, and verbal working memory were associated with reduced GM volumes in the frontal lobe particularly DLPFC, insula, and thalamus, new learning with right superior and middle frontal gyrus, immediate and delayed recall with temporal lobe, cingulate gyrus, caudate and cerebellum. MSA patients showed widespread atrophy involving frontotemporal cortical areas, insula, caudate, thalamus, and cerebellum which correlated with impaired attention/execution, verbal memory, new learning, and memory. Our study results provide evidence to the hypothesis that cognitive impairment in MSA is due to multiregional degeneration and the functional disruption of the cortico-striatal circuit by primary cortical, cerebellar, or thalamic pathology. The study's limitations were the lack of longitudinal cognitive assessment and MRI follow-up of the MSA patients to look for the development of frank dementia. We had not evaluated the contribution of orthostatic hypotension and nocturnal hypoxia due to sleep-disordered breathing towards cognitive deficit in MSA. The findings from this study need to be proved in a larger cohort of MSA patients.

## Conclusions

This study showed evidence of cognitive deficits in all cognitive domains tested in patients with MSA. The impaired cognitive domains had a significant correlation with atrophy of frontotemporal cortical areas, insula, caudate, thalamus, and cerebellum. In MSA, cognitive deficits occur, and impaired cognition is due to the pathology in both cortical and subcortical structures. Long-term follow-up studies are required to find out the progression of these cognitive changes.

## Appendices

**Table 5:** Areas of brain showing significant negative correlation with Stroop effect. * No Brodmann area defined for this part

X	Y	Z	peak equivalent	peak p	Brain area	X
47.06	-3.01	43.97	3.685	0.717405	Right precentral gyrus	Area 6
3.09	-14.5	4.31	3.609	0.717405	Right thalamus	Medial Dorsal Nucleus
7.15	-15.3	12.41	3.314	0.717405	Right thalamus	Medial Dorsal Nucleus
42.06	5.26	2.78	3.51	0.717405	Right insula	*

**Table 6:** Areas of brain showing significant positive correlation with animal naming test results.

X	Y	Z	peak equivalent	peak p	Brain area	Brodmann area
-9.02	11.68	-17.74	4.075	0.972	Left middle frontal gyrus	Area 8
57.01	1.43	27	3.177	0.972	Right Precentral Gyrus	Area 6
13.46	-72.1	44.97	3.112	0.9728	Right Precuneus	Area 7

**Table 7:** Areas of brain showing significant positive correlation with digit span forward scores.

X coor	Y coor	Z coor	peak equivalent	peak p	Cerebrum	Area	Brodmann area
-26.33	35.75	39.65	3.13	0.99	Left frontal lobe	Middle Frontal Gyrus	Area 8

**Table 8:** Areas of brain showing significant positive correlation with total correct response in AVLT test. AVLT-auditory verbal learning test

X coor	Y coor	Z coor	peak equivalent	peak p	Cerebrum	Brodmann area
12.45	28.04	44.98	3.823	0.651	Right Middle Frontal Gyrus	Area 8
19.49	41.1	40.93	3.641	0.651	Right Superior Frontal Gyrus	Area 8
-11.6	-14.97	67.52	3.199	0.718	Left Superior Frontal Gyrus	Area 6
-11.39	-31.18	45.72	3.109	0.772	Left Paracentral Lobule	Area 5

**Table 9:** Areas of brain showing significant positive correlation with immediate recall scores. *No Brodmann area defined for this part

X coor	Y coor	Z coor	peak equivalent	peak p	Brain	Lobe	Area	Brodmann area
28.47	-43.24	-34.4	3.736	0.574	Right Cerebellum	Posterior Lobe	Cerebellar Tonsil	*
-11.84	-11.93	-21.3	3.675	0.574	Left Cerebrum	Limbic Lobe	Uncus	Area 34
-24.71	-57.15	-5.61	3.552	0.574	Left Cerebrum	Limbic Lobe	Parahippocampal Gyrus	Area 19
-14.77	-11.7	-9.24	3.292	0.574	Left Cerebrum	Limbic Lobe	Parahippocampal Gyrus	Area 28
15.88	-9.68	-16.6	3.234	0.574	Right Cerebrum	Limbic Lobe	Parahippocampal Gyrus	Area 34

**Table 10:** Areas of brain showing significant positive correlation with delayed recall score.

X coor	Y coor	Z coor	peak equivalent	peak p	Cerebrum	Area
9.96	9.58	17.51	3.697	0.613	Right Cerebrum	Caudate body
5.93	10.64	6.74	3.198	0.613	Right Cerebrum	Caudate body
-10.89	6.76	18.25	3.658	0.613	Left Cerebrum	Caudate body
-1.47	-9.23	37.16	3.317	0.613	Left Cerebrum	Cingulate Gyrus (Area 24)
-14.77	-13.1	-9.37	3.174	0.613	Left Cerebrum	Parahippocampal Gyrus (Area 28)
